# The Value of Shear Wave Elastography in the Diagnosis of Breast Cancer Axillary Lymph Node Metastasis and Its Correlation With Molecular Classification of Breast Masses

**DOI:** 10.3389/fonc.2022.846568

**Published:** 2022-03-17

**Authors:** Changyun Luo, Li Lu, Weifu Zhang, Xiangqi Li, Ping Zhou, Zhangshen Ran

**Affiliations:** ^1^ Regular Physical Examination Centre, The Second Affiliated Hospital of Shandong First Medical University, Taian, China; ^2^ Ultrasonography Department, The Second Affiliated Hospital of Shandong First Medical University, Taian, China; ^3^ Public Health Section, The Second Affiliated Hospital of Shandong First Medical University, Taian, China; ^4^ Breast Surgery, The Second Affiliated Hospital of Shandong First Medical University, Taian, China; ^5^ Liyang People’s Hospital, Liyang, China

**Keywords:** shear wave elastography, breast cancer, metastasis, molecular classification, diagnosis

## Abstract

**Objective:**

To explore the diagnostic value of shear wave elastography examination (SWE) on axillary node metastasis (ANM) in breast cancer, this study aimed to evaluate the correlation between the SWE features and different molecular types of breast cancer, and to check the elastic modulus differences among the molecular types.

**Methods:**

Breast cancer patients from November 2020 to December 2021 were subjected to both conventional ultrasonic examination (CUE) and SWE before ultrasound-guided percutaneous biopsy or axillary lymph node dissection (ALND). We used the pathological results as the gold standard to draw the receiver operating characteristic (ROC) curve.

**Results:**

SWE outperforms CUE, but their conjunctive use is the best option. No significant correlation was found between the elastic modulus values and the molecular types of breast cancer.

**Conclusion:**

SWE can be used as an routine auxiliary method of CUE for ANM.

## Introduction

In the 21st century, cancer is the top cause of death in hospitals and the key limitation of life expectancy in most countries, whatever level their economics and social civilization (1, 2). The Global Cancer Statistics Report published by the United States Cancer Research Institute shows that breast cancer surpassed lung cancer in 2020, becoming the most common type of cancer among female patients and the main cause of death for female patients in 185 countries (3). According to statistics, 19.3 million people were diagnosis with cancer and nearly 10 million deaths worldwide in 2020 were because of cancer (4). Around 2.3 million new-onset cases are of female breast cancer accounting for 11.7%, and breast cancer causes 0.69 million deaths which account for 6.9% of all global cancer deaths (5). In fact, 1 in every 18 women will develop breast cancer globally, and the clinical manifestations and prognosis of patients are different (6). Age, molecular subtype, and axillary lymph node status are considered to be independent factors affecting the prognosis of patients suffering from breast cancer (7). In addition, tumor-related factors such as pathological type, grade, and stage can also explain the higher mortality of breast cancer to a certain extent (8).

With the development of molecular biology, it has been recognized that breast cancer has large biological diversity and high heterogeneity, which result in different morphological subtypes, recurrence rate, targeted therapy strategies, and survival risks (9–11). Therefore, if the patients with breast cancer can be accurately classified, it should help to select individualized precision treatment and effectively predict the prognosis (12–14). According to immunohistochemical indexes such as estrogen receptor (ER), progesterone receptor (PR), proliferating cell nuclear antigen (Ki-67), and human epidermal growth factor receptor-2 (HER-2), clinicians determine the molecular subtype of breast cancer, namely luminal A, luminal B, Her-2-positive, and triple-negative breast cancer (TNBC) (15–18), where HER2-positive includes HR-negative and HR-positive (15, 19–23). The status of axillary lymph nodes is also an important factor influencing the prognosis of patients. According to reports, 70-80% of early non-metastatic breast cancer patients can be cured. Patients with advanced breast cancer and distant organ metastasis are considered to be incurable by existing therapies. The prognosis of patients with advanced breast cancer is poor, and the 5-year survival rate is only 20% (24). In addition, the axillary lymph node (ALN) is deemed as the first site to be metastasized by breast cancer through the lymphatic vessels (25).

Shear wave elastography examination (SWE) is a newly emerging elastography technique, which can display tissue stiffness in a quantified form to obtain the biological information of the primary lesion (26–30). At present, many studies have verified the diagnostic value of SWE for benign and malignant lesions in breasts (31–34). The technique has been widely employed to check the thyroid, pancreas, kidney, prostate, liver, and other organs while few studies about axillary node metastasis (ANM) and its application for the molecular classification of breast cancer were reported (35–39). Here, we applied SWE to assess the axillary node status of patients with breast cancer with a goal to explore its feasibility in the diagnosis of ANM, and to check the relationship of the SWE elastic modulus and the molecular types of breast cancer.

## Materials and Methods

### Research Objects

After the pathological verification for breast cancer, 114 patients who had never received any treatment were recruited in the Second Affiliated Hospital of Shandong First Medical University from November 2020 to December 2021 (40). The mean age is 52.52 ± 9.03 (range, 31-75 years old), and the mean long diameter of the lymph node is 1.60 ± 0.70 (range, 0.5 ± 4.8 cm). All of the patients underwent conventional ultrasonic examination (CUE) before ultrasound-guided percutaneous biopsy on the axillary lode or axillary lymph node dissection (ALND). Some key indexes were scored (see **Table 1**) . This program was approved by the Medical Ethics Committee of the Second Affiliated Hospital of Shandong First Medical University.

**Table 1:** Criteria and evaluation of CUE in the diagnosis of lymph node status.

Index	1 point	2 point
Aspect ratio	>2	<2
Short axis diameter	<7 mm	>7 mm
Lymphatic hilus	Yes	No
Cortical thickness	<3 mm	>3 mm
Blood flow type	Gate type	Peripheral type or mixed type

### Shear Wave Elastography Examination

The Toshiba Apio500 ultrasonic diagnostic machine equipped with high-frequency linear array probe PLY-805AT (2.0-12.0 MHZ) called shear wave was used for SWE. Based on the operations of Skerl et al., the parameters of SWE were set when ROI = 2 mm (41). Both of the transverse and the longitudinal sections of each breast mass and suspicious lymph node were measured three times to obtain average values.

### Image Analysis

Two physicians who have more than 5 years of experience in breast and axillary lymph node diagnosis analyzed the image results. A score was evaluated based on the aspect ratio and the short axis diameter of the lymph node (42). Plus, the maximum value (Emax), average value (Emean), and minimum value (Emin) of Young’s modulus were assessed by SWE, and a static image was kept (43, 44). Afterwards, univariate analysis was performed using ALN metastasis as a dependent variable and the CUE scores. The obtained indexes with statistical significance were extracted for multivariate logistic regression analysis with the Emean of SWE as independent variables. In the predictive model, ANMs were confirmed as benign or malignant lesions. Then, the predictive results were compared with the below pathological results to draw the gold standard receiver operating characteristic (ROC) curves and get the area under the curve (AUC) values.

The patients who underwent surgical treatment were classified into four groups according to their molecular classification results to check whether the elastic modulus values of SWE ​​were statistically different between the groups, and explore its relationship with the classification strategy.

### Pathological Examination

Ultrasound-guided axillary nodal puncture was accomplished in 62 patients and breast mass resection was done in 86 patients. Tissue biopsy including postoperative pathological section and immunohistochemical examination was implemented (45, 46).

### Statistical Analysis

SPSS 25.0 software was used to process the above data. The measured data were expressed as mean ± standard deviation, and the count data were expressed as rate (%). The chi-square test was used to compare the two-category data between the two groups, and the KAPPA test was used to compare the consistency of the diagnosis results of CUE, SWE, and their conjunctive usage with the pathological results. Multivariate logistic regression analysis was used to construct a prediction model of CUE combined with SWE to obtain the prediction probability.

## Results

### Pathological Results

A total of 114 women with breast cancer were enrolled in this study, who were then divided into two groups: the ANM group with 58 cases and the non-metastasis group with 56 cases. The mean age, medical course, and ALN size in the former group were 49.59 ± 8.54 years, 11.0 ± 25.22 months, and 2.16 ± 2.63 cm. In the latter group, the same data were 51.48 ± 9.50 years, 5.70 ± 14.27 months, and 1.36 ± 0.51 cm.

### Comparison of the Two Groups With/Without ANM

As **Table 2** shows, on one hand, the CUE comparison displayed significant differences in lymph nodal size, aspect ratio, and short axis diameter. However, the hilum structures, cortex thicknesses, and blood-flow types did not show any difference. On the other hand, the SWE comparison also showed significant differences in the Emax, Emean, and Emin values.

**Table 2:** Comparison of CUE and SWE elastic modulus between the two groups with/without ANM.

	No lymph node metastases	Lymph node metastases	T/X^2^	p value
Lymph node size	1.36 ± 0.51	2.16 ± 2.63	-2.218	0.029
Aspect ratio >2	41 (73.2%)	13 (22.4%)		
<2	15 (26.8%)	45 (77.6%)	29.49	0
Short axis diameter <7	44	19	24.18	0
>7	12	39		
Lymphatic hilus	26	26	0.029	0.864
No lymphatic hilus	30	32		
Gate type	28	21	2.21	0.137
Not gate type	28	37		
Cortical thickness <3	11	13	0.13	0.717
>3	45	45		
Emax	24.68 ± 18.91	77.68 ± 48.06	-7.693	0
Emean	17.34 ± 14.13	58.33 ± 42.31	-6.887	0
Emin	13.35 ± 10.39	39.77 ± 36.79	-5.255	0

### Comparison of the Consistency Between the Diagnosis Results of the Three Medical Means and the Pathological Outcomes

In the CUE assessment for ANM, as **Table 3** shows, compared with the pathological results, the accuracy, sensitivity, specificity, positive predictive value, and negative predictive value calculated from the exclusive usage of CUE in the diagnosis of malignant lymph nodes were 65.8%, 72.4%, 58.9%, 64.6%, and 67.3%, respectively. As a result, the whole consistency with the pathological results was 0.314.

**Table 3:** Comparison of diagnostic efficacy of CUE, SWE, and their conjunctive use in ANM.

	Sensitivity	Specificity	Positive predictive value	Negative predictive value	Accuracy	Kappa
CUE	72.40%	58.90%	64.60%	67.30%	65.80%	0.314
SWE	70.70%	76.70%	75.90%	71.60%	73.60%	0.474
CUE+SWE	79.30%	82.10%	82.10%	79.30%	80.70%	0.616

In the SWE assessment for ANM, according to the literature, Emean>18.7 was set as the metastatic threshold of the lymph nodes. When the average stiffness of the lymph node was greater than 18.7 Kpa, we believed that the lymph node was more likely to be malignant. On the contrary, the lymph node was more likely to be benign. Compared with the pathological results, the sensitivity, specificity, and positive and negative predictive value of SWE for malignancy were 70.7%, 76.7%, 75.9%, and 71.6%, respectively, which resulted in a diagnostic accuracy of 73.6%, and the consistency with the pathological result was 0.474.

In the conjunctive use of CUE and SWE to assess ANM, whether metastasis exists was used as the dependent variable and the indicators observed by CUE were used as independent variables, and univariate analysis was performed. It was found that the aspect ratio and short axis diameter of lymph nodes observed by CUE were statistically significant with ANM. Then, taking the average elastic modulus of SWE, the aspect ratio of the lymph node, and the short axis diameter of the lymph node as the independent variables into the multivariate logistic regression, a predictive model was constructed. The results show that Emean and both the aspect ratio of the lymph node and the short axis diameter of the lymph node can be entered into the equation.

**Figure 1:**
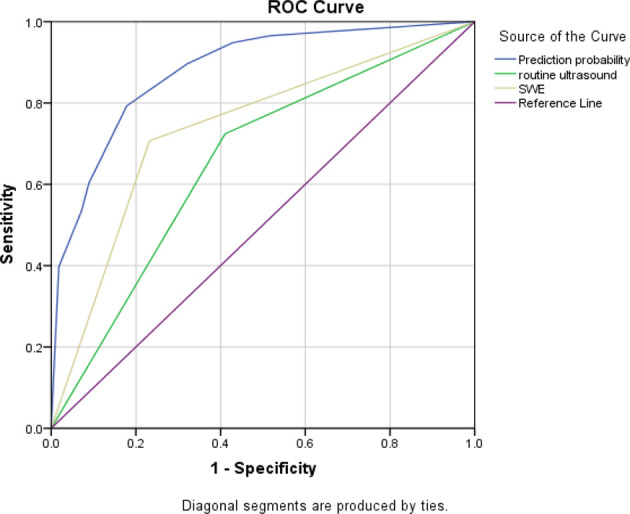
Three ROC curves for the sensitivity and specificity of CUE, SWE, and their conjunctive use in ANM diagnosis.

Based on the above results, three ROC curves were drawn and the corresponding AUC values were calculated (see **Figure 1**).The results showed that conjunctive use (AUC, 0.88) had the best predictive ability compared to exclusive use of CUE (AUC, 0.657) or SWE (AUC, 0.737).

### The Best Cut-Off Value of SWE for the Diagnosis of ANM

**Figure 2:**
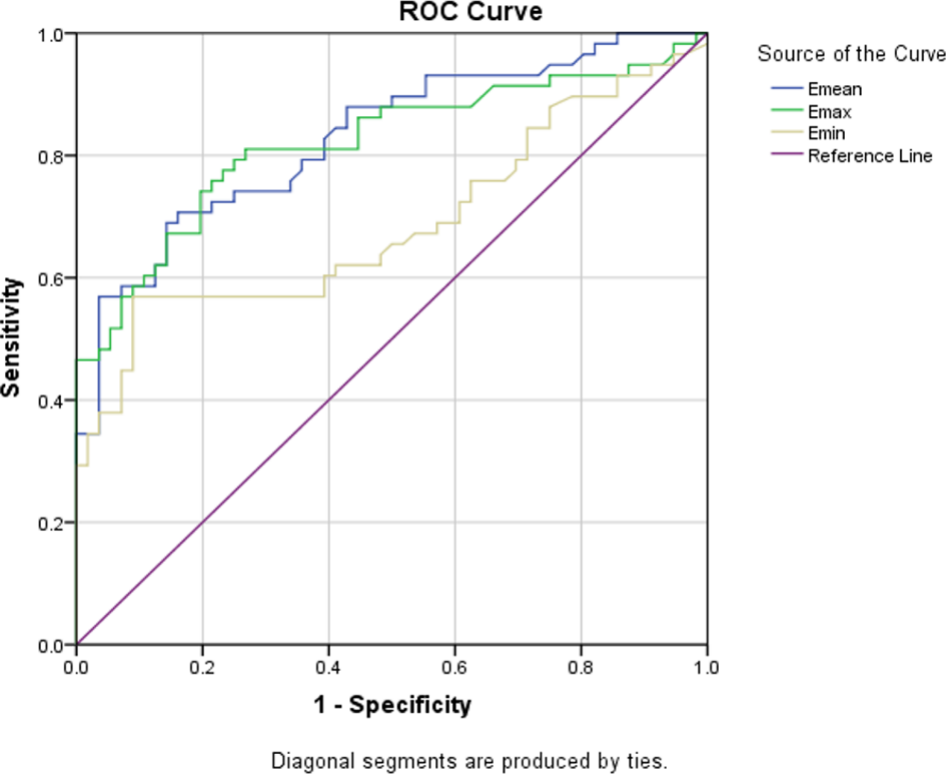
Three ROC curves for the sensitivity and specificity of the Emean, Emax, and Emin in the application of ANM diagnosis.

Although many studies have shown that quantitative SWE can help diagnose breast diseases, the cut-off values used were different. In order to evaluate the optimal SWE parameters to quantify ANM, ROC curves for Emax, Emean, and Emin were also drawn. We suggest that when Emean=23.2 is used as the cut-off value, SWE is the best (see **Figure 2**).

### The SWE Elastic Modulus Value and Analysis of Variance Results of Different Molecular Types of Breast Cancer

According to the expression of ER, PR, HER-2, and Ki-67, 86 people who underwent surgery were divided into six groups (19, 47). Variance analysis on the elastic modulus values for each group was performed. We found that all of the comparisons of Emax, Emean, and Emin between the molecular types did not have statistical significance (see **Table 4**).

**Table 4:** Variance analysis of SWE elastic modulus and different molecular subtypes of breast cancer.

	Emax (Kpa)	Emean (Kpa)	Emin (Kpa)
Luminal A type	119.04 ± 17.63	88.07 ± 23.02	59.53 ± 24.19
Luminal B type	112.43 ± 14.62	80.97 ± 11.65	51.07 ± 10.29
HER-2 high expression	119.51 ± 21.60	89.19 ± 21.03	60.50 ± 20.82
Triple-negative	126.87 ± 16.45	98.14 ± 17.53	68.90 ± 16.53
F	1.372	1.714	1.719
P	0.257	0.171	0.169

## Discussion

Worldwide, the incidence and mortality of breast cancer always takes the top cancer spot in female patients (3). Several reports show that the incidence of breast cancer has been increasing year by year in the past 5 years (48, 49), and the choice of treatment strategy is determined by the state of ALNs, which decide the final bill of the patients (50). Therefore, it is substantially important to correctly assess the status of ALNs in breast cancer patients before surgery.

Although CUE has high sensitivity in the diagnosis of breast cancer, some previous studies have found that the accuracy, sensitivity, and specificity of ultrasound in diagnosing ALN metastasis is not so high (51–53). The concept of SWE was first proposed by Sarvazyan et al. in 1998 (54). Its principle is to use acoustic radiation force pulses (ARFI) to apply pressure to the tissues to induce mechanical vibrations in the tissues. In the process, by collecting the shear echo signal reflected by the tissue vibration, the propagation speed in the tissue can by calculated and automatically converted into the elastic modulus value through the conversion system. Consequently, the hardness information of the tissue can be quantified (55). Nowadays, a large number of studies have shown that the advantages of SWE in the application of breast, thyroid, prostate, liver, and other organs, but no research has reported the assessment of ALN metastasis. Besides, there is no standard for the cut-off value of SWE in the diagnosis of metastatic lymph nodes.

In this study, the Emax, Emean, and Emin of the lymph node metastasis group were all higher than those of the lymph node non-metastasis group, and the difference was statistically significant (p<0.05). The average elasticity of benign and malignant lymph nodes was 17.34 Kpa and 58.33 Kpa, respectively. The average value we obtained was higher than some previous studies, which may be longer than the course of some breast cancer patients in our study. The tumor cells synthesize a large number of collagen fibers and lymphocytes in the tumor microenvironment during the process of metastasis. Infiltration changes increased the stiffness of the lymph nodes in this part of the patients, leading to a corresponding increase in the average stiffness of the lymph node metastasis group. In this study, Emean=18.7 kpa was selected as the critical value of metastatic lymph nodes. A preliminary exploration was carried out on shear wave elastography to assess lymph node metastasis. A total of 114 lymph nodes were examined by shear wave elastography, and 41 cases of metastatic lymph nodes were correctly diagnosed, which were benign. There were 43 cases of lymph nodes and 30 cases of misdiagnosis. Its specificity (76.7%) and accuracy (73.6%) were higher than those of conventional ultrasound, but its sensitivity (70.7%) was lower, so it could not be used as a substitute for conventional ultrasound. The reasons for the misdiagnosis included: 1) It may be because the volume of some lymph nodes is relatively small or the location is relatively deep. Affected by the anatomical structure of the axilla, the shear wave cannot spread well, resulting in a low measured elastic modulus value. 2) There may be liquefaction and necrosis in some malignant lymph nodes, and there are relatively few elastic and collagen fibers in them, so the measured elastic modulus value is not high.

Additionally, as we believe that the average stiffness of metastatic lymph nodes has the highest specificity, the conjunctive use of Emean and CUE can form complementary advantages, obtaining more objective information to determine which lymph nodes are suitable for biopsy. However, up to now, the optimal cut-off value of each parameter of SWE has not yet reached agreement. It may be affected by many factors, such as pre-compression, the machine model and the depth of the lesion, and the progression of the patient’s disease (56, 57). Therefore, studies with a larger sample size involving multiple units should be considered. Plus, we tried to employ SWE to predict the molecular type of breast cancer with the elastic modulus values (58, 59). Unfortunately, no significant difference was found ​​between the six different groups. This is in line with the conclusion drawn by previously published papers (60, 61). As a result, we do not recommend the implementation of the molecular classification of breast cancer *via* SWE at this stage. However, with the increase of clinical experience, doctors have gradually realized the value of SWE in the diagnosis of breast cancer axillary lymph node metastasis (62–65).

## Data Availability Statement

The original contributions presented in the study are included in the article/supplementary material. Further inquiries can be directed to the corresponding author.

## Ethics Statement

The study was approved by the Medical Ethics Committee of the Second Affiliated Hospital of Shandong First Medical University.

## Author Contributions

ZSR designed the study. WZ and XL recruited the patients and summarized their medical records. CL and LL performed the data analysis and wrote the manuscript. PZ revised the manuscript. All the authors made a direct and intellectual contribution to this topic and approved the article for publication.

## Funding

This work was supported by grants from the Shandong Province Traditional Chinese Medicine Science and Technology Development Plan (No. 2015-266); the Shandong First Medical University Academic Improvement Plan (No. 2019QL017); the Shandong Province Medicine and Health Science and Technology Development Plan (No. 202009020793); the Shandong Province Natural Science Foundation (No. ZR2020MH357); and the Tai’an Science and Technology Innovation Development Project (No. 2020NS205).

## Conflict of Interest

The authors declare that the research was conducted in the absence of any commercial or financial relationships that could be construed as a potential conflict of interest.

## Publisher’s Note

All claims expressed in this article are solely those of the authors and do not necessarily represent those of their affiliated organizations, or those of the publisher, the editors and the reviewers. Any product that may be evaluated in this article, or claim that may be made by its manufacturer, is not guaranteed or endorsed by the publisher.
